# TGF-β Receptor Inhibitor SB431542 Enhanced the Sensitivity of Gastric Cancer to 5-Fluorouracil: New Combined Targeted Therapy

**DOI:** 10.3390/ijms262311250

**Published:** 2025-11-21

**Authors:** Sara Bonomo, Roberto Giovannoni, Marialuisa Lavitrano, Massimiliano Cadamuro, Donatella Conconi

**Affiliations:** 1 School of Medicine and Surgery, University of Milano-Bicocca, Via Cadore 48, 20900 Monza, Italy; sara.bonomo@unimib.it (S.B.); massimiliano.cadamuro@unimib.it (M.C.); donatella.conconi@unimib.it (D.C.); 2 Department of Biology, University of Pisa, Via Derna 1, 56126 Pisa, Italy; roberto.giovannoni@unipi.it

**Keywords:** TGF-β, gastric cancer, TGFBR1, 5FU, chemoresistance, apoptosis

## Abstract

Gastric cancer (GC) continues to be a major cause of cancer-related deaths globally, primarily due to resistance to standard treatments like 5-fluorouracil (5FU). The transforming growth factor-β (TGF-β) signaling pathway is recognized as a key contributor to tumor progression and resistance to therapy. This work investigated the therapeutic potential of targeting TGF-β receptor I (TGFBR1) with the selective inhibitor SB431542 to enhance the effect of 5FU in GC. Analysis of public gene expression datasets revealed that increased levels of TGF-β and TGFBR1 are significantly connected with poor prognosis, particularly in high-grade GC. In vitro experiments using AGS and SNU-1 cell lines demonstrated that co-treatment with SB431542 and 5FU significantly reduced cell viability, making GC cells more sensitive to 5FU. This combination treatment led to a significant activation of caspase-dependent apoptosis, indicating an enhanced pro-apoptotic effect. These findings suggest that TGFBR1 inhibition could provide a strategic approach to reduce the dosage of 5FU, thereby minimizing its severe side effects in gastric cancer patients. Furthermore, these results underscore the potential of TGFBR1 as both a prognostic biomarker and a therapeutic target, warranting further investigation in aggressive forms of gastric cancer.

## 1. Introduction

Gastric cancer (GC) represents a significant global health challenge, ranking as the fifth most common malignancy and the fifth leading cause of cancer-related mortality worldwide [1]. Its incidence shows a marked gender disparity, occurring in men at nearly twice the rate observed in women [1,2]. Patient outcomes are generally poor, with an average 5-year survival rate of only 20–30% from the time of diagnosis, largely due to delayed detection. In its early stages, this cancer tends to be insidious, presenting with vague and nonspecific symptoms [2]. However, early diagnosis could increase patient survival at 5 years by up to 90% [3]. The pathogenesis of GC is multi-factorial and characterized by multiple stages. Early GC are usually confined to the mucosa or submucosa, while advanced GC are characterized by spreading beyond the submucosa to infiltrate nearby organs and are prone to metastasis and burdened by a low overall survival of the patients [4,5].

Treatment options for GC include surgery, chemotherapy, radiotherapy, and targeted therapies such as monoclonal antibodies [6]. The choice of therapy depends on the cancer stage and the patient’s overall health status. Surgery—often the initial approach—may involve partial or total gastrectomy combined with lymph node dissection. Chemotherapy can be administered before (neoadjuvant), after (adjuvant), or as the primary treatment for inoperable tumors [7]. Radiation, sometimes combined with chemotherapy, helps shrink tumors or alleviate symptoms in advanced stages [8]. Targeted therapies, including monoclonal antibodies, are used alongside chemotherapy for HER2-positive tumors [9]. However, the effectiveness of current therapies is often limited, particularly in advanced stages. This highlights the need for a better understanding of the molecular mechanisms of GC, which could lead to more effective and personalized treatment approaches.

Transforming growth factor (TGF) β is a key mediator in the development and malignant progression of GC, especially in its advanced stages [10]. TGF-β is a pleiotropic cytokine secreted by a wide number of cell types, which is particularly important in tumorigenesis, supporting invasion and metastasis of neoplastic cells by modulating the immune system and tumor microenvironment [11]. Furthermore, it has been recently demonstrated that TGF-β overexpression under VEGF-A stimulation is able to induce multidrug resistance (MDR) in GCs [12]. For all these reasons, the signal pathways modulated by this cytokine results in a very attractive therapeutic target. The most advanced therapeutic approach, in recent years, is linked to the use of drugs that target specific signaling pathways and therefore the manipulation of the pathway supervised by TGF-β could give excellent results in terms of efficacy, given the biological importance of this growth factor. Thus, the aim of this manuscript is to investigate whether manipulation of the TGF-β signaling pathway, using a specific inhibitor, is able to enhance sensitivity to chemotherapeutic drugs in different GC cell lines.

## 2. Results

### 2.1. TGF-β and TGFBR1 Expression Correlate with Gastric Cancer Patients’ Prognosis

**Figure 1:**
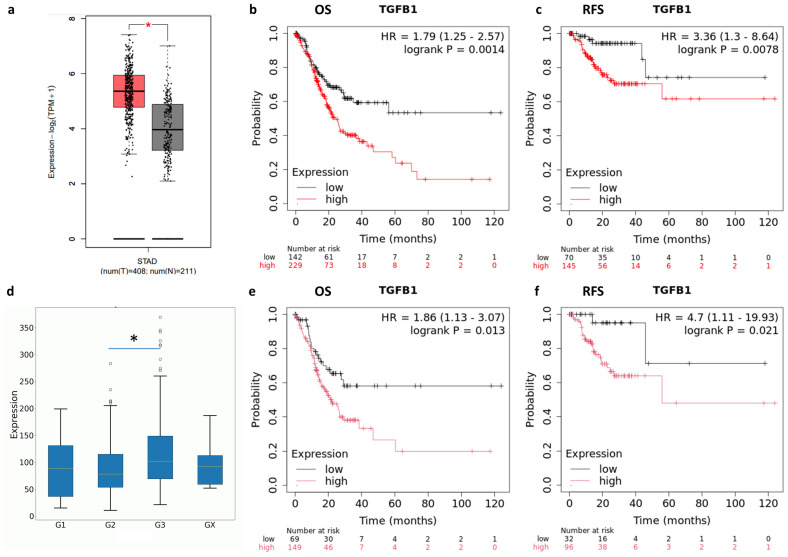
(**a**) GEPIA2 analysis of TGF-β expression in the TCGA-STAD cohort (red box) versus TCGA normal and GTEx data (grey box) (*p* < 0.01, red five pointed star). Kaplan–Meier Plotter correlation analysis between patients’ overall survival (OS) (**b**) or relapse-free survival (RFS) (**c**) and TGF-β expression levels in TCGA-STAD dataset. The log-rank test was used to compare the groups; *p*-value < 0.05 was considered statistically significant. (**d**) TGF-β expression in relation to histological grade (*p* < 0.01, black asterisk). G1: Well-differentiated (low grade); G2: Moderately differentiated (intermediate grade); G3: Poorly differentiated (high grade); Gx: Grade cannot be assessed (undetermined). Kaplan–Meier Plotter correlation analysis between patients’ OS (**e**) or RFS (**f**) and TGF-β expression levels in grade 3 TCGA-STAD dataset. The log-rank test was used to compare the groups; *p*-value < 0.05 was considered statistically significant. HR: Hazard Ratio.

To explore TGF-β expression in the TCGA-STAD cohort (The Cancer Genome Atlas-Stomach Adenocarcinoma), several bioinformatics analyses were performed. GEPIA2 analysis revealed a significant upregulation of TGF-β in tumor biopsies compared to normal tissues (*p* < 0.01, Figure 1a). Moreover, high TGF-β expression in tumor samples was associated with worse overall survival (OS) and relapse-free survival (RFS), supporting its potential role as a prognostic marker in GC progression (Figure 1b,c).

To further assess the relationship between TGF-β expression and clinical parameters, the OncoDB web server was utilized. A statistically significant difference was found between grade 2 and grade 3 tumors (*p* < 0.01, Figure 1d). Due to the limited number of grade 1 samples (*n* = 12), no statistically significant conclusions could be drawn for this subgroup. Finally, the correlation between TGF-β expression and prognosis was specifically examined in grade 3 tumors (Figure 1e,f). These data confirmed the pivotal role of TGF-β expression GC prognosis, especially in the higher-grade subgroup.

**Figure 2:**
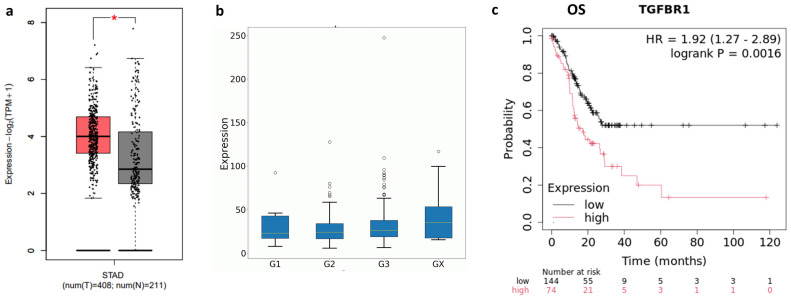
(**a**) GEPIA2 analysis of TGFBR1 expression in the TCGA-STAD cohort (red box) versus TCGA normal and GTEx data (grey box) (*p* < 0.01, red five pointed star). (**b**) TGFBR1 expression in relation to histological grade. G1: Well-differentiated (low grade); G2: Moderately differentiated (intermediate grade); G3: Poorly differentiated (high grade); Gx: Grade cannot be assessed (undetermined). (**c**) Kaplan–Meier Plotter correlation analysis between patients’ OS and TGFBR1 expression levels in grade 3 TCGA-STAD dataset. The log-rank test was used to compare the groups; *p*-value < 0.05 was considered statistically significant. HR: Hazard Ratio.

Canonical TGF-β signaling occurs when the ligand binds to TGFBR2 (Transforming Growth Factor β receptor II), which in turn recruits and phosphorylates TGFBR1 (TGF-β receptor I). Activated TGFBR1 then phosphorylates the downstream effectors SMAD2 and SMAD3 [13]. Therefore, we evaluated the expression of both receptors in TCGA-STAD cohort. GEPIA2 analysis showed a significant upregulation of TGFBR1 in tumor biopsies compared to normal tissues (*p* < 0.01, Figure 2a). Moreover, although TGFBR1 expression did not correlate with histological grading, a significant correlation was observed between its expression and OS in grade 3 patients (Figure 2b,c). These data suggested the prognostic role of TGFBR1 in high-grade GC.

### 2.2. Inhibition of TGF-β Signaling Modulates Both Cell Viability and Proliferation in GC Cell Lines

**Figure 3:**
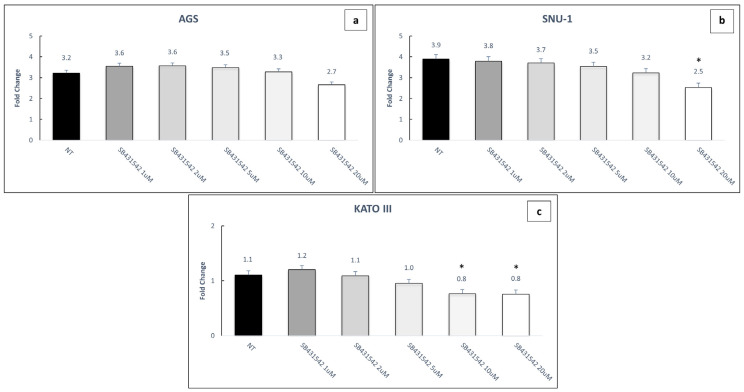
Cell viability experiments with SB431542. AGS (**a**), SNU-1 (**b**) and KATO III (**c**) fold change in cell viability after 72 h treatments with increasing doses of SB431542. Cell viability was assessed by Cell Titer Glo Assay. NT: untreated cells. The graphs represent the average of three separate experiments. Mean ± SEM are plotted in the graphs. *p*-value < 0.05 was considered statistically significant (* *p* < 0.05 vs. NT).

Based on previous data, we treated three poorly differentiated GC cell lines, AGS, SNU-1 and KATO III [14,15,16] with increasing concentration of SB431542 (0, 1, 2, 5, 10, 20 μM), a selective inhibitor of TGFBR1, for 72 h. These cells carried distinct genetic mutations in the TGF-β pathway, resulting in differential pathway activation (Figure S1). The results showed a dose-dependent effect and, in particular, higher concentrations (10, 20 μM) significantly reduced cell viability in SNU-1 and KATO III cells compared to untreated controls, whereas in AGS cells the reduction was not statistically significant (Figure 3a–c).

**Figure 4:**
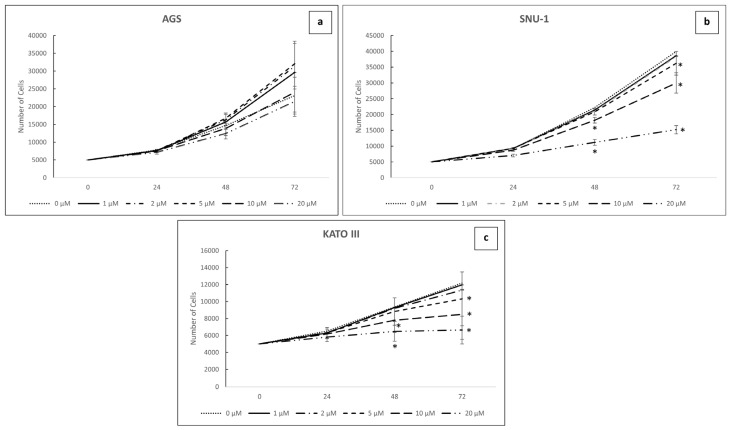
Cell proliferation experiments with SB431542. AGS (**a**), SNU-1 (**b**) and KATO III (**c**) number of cells after treatments with increasing doses of SB431542 (0, 1, 2, 5, 10, 20 μM). Cell proliferation was assessed by Cell Titer Glo Assay every 24 h for 72 h. The graphs represent the average of three separate experiments. Mean ± SEM are plotted in the graphs. *p*-value < 0.05 was considered statistically significant (* *p* < 0.05 vs. NT).

Similarly, a significant reduction in cell proliferation was obtained upon evaluating cell proliferation using the same doses of SB431542 at different time-points (24, 48 and 72 h). Notably, in SNU-1 and KATO III cells, proliferation was significantly reduced after 48 h of treatment with 10 and 20 μM, and after 72 h with 5, 10 and 20 μM of the inhibitor (Figure 4a–c). Proliferation in AGS cells was not affected by SB431542 at any dose. These findings confirm that TGF-β signaling plays a key role in the modulation of two major hallmarks of tumor aggressiveness: cell proliferation and viability.

### 2.3. Inhibition of TGF-β Signaling Pathway Enhances the Toxic Effect of 5-Fluorouracil on AGS and SNU-1 Cell Lines

To verify whether the toxic effect of treatment with 5-fluorouracil (5FU), a widely used chemotherapeutic in GC, is exacerbated by the inhibition of the TGF-β signaling pathway, we continued the experiments with AGS and SNU-1, the two cell lines resulted more resistant to the toxic effect of SB431542 (the corresponding experiments on KATO III are shown in Figures S2 and S3). In these cell lines, in fact, treatment with SB431542 did not show a toxic effect and therefore any increase in toxicity would be due solely to its combined treatment with 5FU.

**Figure 5:**
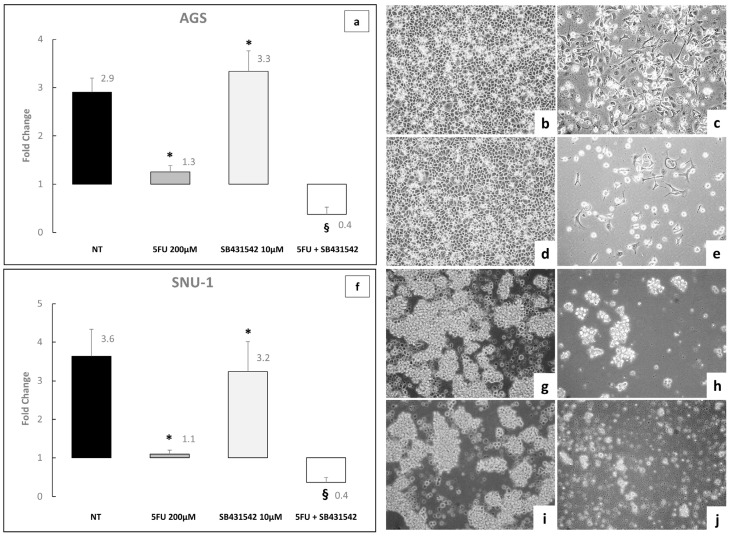
Cell viability experiments with 5FU (200 μM) or SB431542 (10 μM) alone or in combination on AGS (**a**) and SNU-1 (**f**) cells. Cell viability was assessed by the Cell Titer Glo Assay after 72 h of treatments. NT: untreated cells. The graphs represent the mean of three separate experiments. Mean ± SEM are plotted in the graphs. (**b**–**e**) Representative images of AGS cells after 72 h of treatment: (**b**) NT, (**c**) 5FU, (**d**) SB431542, (**e**) 5FU + SB431542. (**g**–**j**) Representative images of SNU-1 cells after 72 h of treatment: (**g**) NT, (**h**) 5FU, (**i**) SB431542, (**j**) 5FU + SB431542. Magnification: 10× for all figures. *p*-value < 0.05 was considered statistically significant (* *p* < 0.05 vs. NT; § *p* < 0.05 vs. both NT and 5FU).

As expected, the treatment with 5FU 200 μM, induced a marked reduction in cell viability on AGS and SNU-1 cells. Notably, co-treatment with SB431542 at 10 μM for 72 h led to a significantly greater decrease in cell viability compared to treatment with 5FU alone (*p* < 0.05; Figure 5). These data are consistent with the hypothesis that inhibition of the TGF-β signaling pathway enhances the efficacy of chemotherapeutic drugs, suggesting that the combined use of 5FU and SB431542 could be an interesting new approach to the treatment of GC.

**Figure 6:**
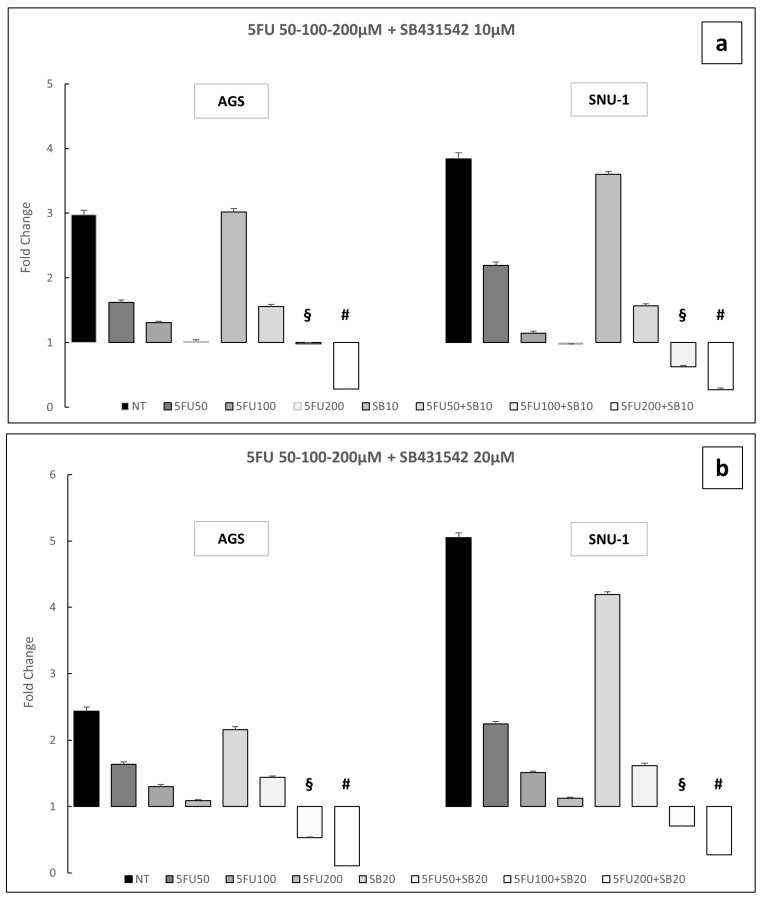
Cell viability experiments with 5FU (50–200 μM) or SB431542 10 μM (**a**) and 20 μM (**b**) alone or in combination on AGS and SNU-1 cells. Cell viability was assessed by the Cell Titer Glo Assay after 72 h of treatments. NT: untreated cells. The graphs represent the mean of three separate experiments. Mean ± SEM are plotted in the graphs. *p*-value < 0.05 was considered statistically significant (§ *p* < 0.05 vs. 5FU 100 μM; # *p* < 0.05 vs. 5FU 200 μM).

In a 72-h toxicity assay, AGS and SNU-1 cells were treated with increasing doses of 5FU (10–200 μM) either alone or in combination with SB431542 10 and 20 μM. We observed that, not only 5FU 200 μM, but also 5FU 100 μM in combination with SB431542 10 and 20 μM was able to reduce cell viability (Figure 6a,b). These findings indicate that inhibition of the TGF-β signaling pathway may enhance the therapeutic efficacy of 5FU, potentially enabling the use of lower chemotherapeutic doses.

### 2.4. Cells Challenged with 5FU or 5FU + SB431542 Died in a Caspase-Dependent Way

**Figure 7:**
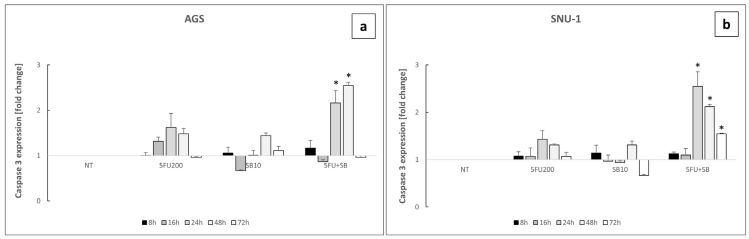
Caspase III-VII assay experiment on AGS (**a**) and SNU-1 (**b**) cells untreated (NT) or treated with 5FU 200 μM, SB431542 10 μM or 5FU 200 μM + SB431542 10 μM at different time points. The graphs represent the mean of three separate experiments. Mean ± SEM are plotted in the graphs. *p*-value < 0.05 was considered statistically significant (* *p* < 0.05 vs. NT).

To verify the involved cell death pathway, we performed a time course experiment at different time points (8, 16, 24, 48, 72 h). The peak signal of caspase activation after combined treatment was reached at 24 h for SNU-1 cells and 48 h for AGS cells followed by a significant decline at later time points (Figure 7a,b).

**Figure 8:**
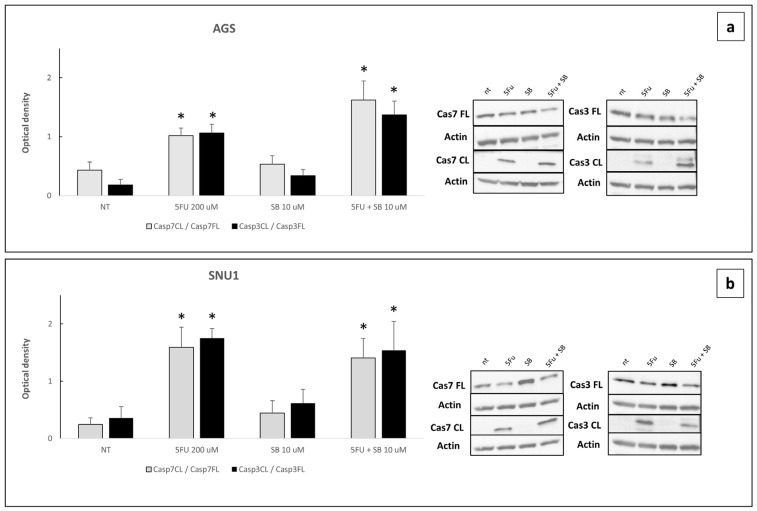
Quantification of Western blot for cleaved (CL) and full length (FL) caspase 3 and caspase 7 in AGS (**a**) and SNU-1 (**b**) cells untreated (NT) or treated with 5FU 200 µM alone or in combination with SB431542 10 µM for 24 h. Value were normalized to the housekeeping protein and the cleaved forms of both were expressed relative to the full-length form. The graphs represent the average of three separate experiments. *p*-value < 0.05 was considered statistically significant (* *p* < 0.05 vs. NT and SB431542 10 μM).

Western blot experiments showed that, after 24 h treatment with 5FU alone or in combination with SB431542, cleaved caspase 3 and cleaved caspase 7 were significantly activates compared to untreated cells or cells treated with SB431542 alone (Figure 8a,b and Figure S4). These data strongly support that the cell death induced by these pharmacological treatments occurs through the apoptotic pathway.

**Figure 9:**
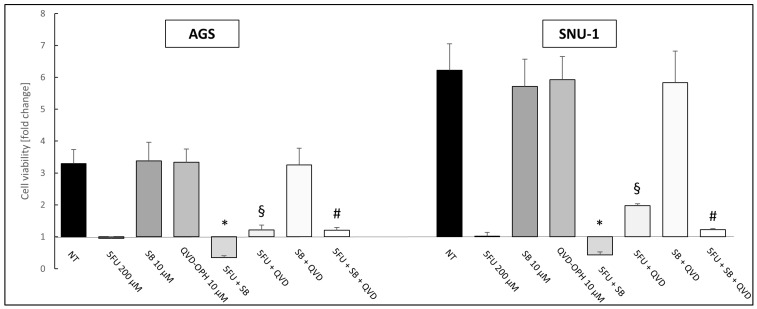
Cell viability experiments with 5FU 200 μM + SB431542 10 μM, 5FU 200 μM + Q-VD-Oph 10 μM and 5FU 200 μM + SB431542 10 μM + Q-VD-Oph 10 μM at 72 h on AGS and SNU-1 cell lines. NT: untreated cells. The graphs represent the mean of three separate experiments. Mean ± SEM are plotted in the graphs. *p*-value < 0.05 was considered statistically significant (* *p* < 0.05 vs. 5FU, § *p* < 0.05 vs. 5FU, # *p* < 0.05 vs. 5FU + SB431542 10 μM).

To further validate the involvement of apoptosis, cells were treated with the pan-caspase inhibitor Q-VD-Oph. In both cell lines, this inhibition led to a significant increase in cell viability (expressed as fold change) compared to treatment with 5FU or in combination with SB431542 (* *p* ≤ 0.05) (Figure 9), confirming the activation of a caspase-dependent cell death.

### 2.5. FU + SB431542 Treatment Is More Effective in Affecting Clonogenicity of AGS Cells Compared to 5FU Alone

To further explore the effect of 5FU alone or in combination with SB431542 on AGS cells proliferation, we performed a clonogenic assay. Following 14 h of treatment with 5FU, SB431542, or their combination, cells were cultured in drug-free medium for 12 days to allow colony formation. Both 5FU and the 5FU + SB431542 treatments significantly reduced colony formation compared to untreated or SB431542 treated cells (Figure S5). Notably, the combination treatment was significantly more effective than 5FU alone in reducing the number of colonies (Figure S5), consistent with the hypothesis that TGF-β signaling inhibition enhances chemosensitivity and counteracts chemoresistance mechanisms.

## 3. Discussion

TGF-β is a multifunctional cytokine which plays several roles in physiology and physiopathology [17,18,19]. In particular, its overexpression has been associated with poor prognosis in different human tumors, including breast, cervical, and colorectal cancers [20,21,22]. In colorectal cancer, TGF-β also contributes to resistance against chemotherapy agents such as 5FU [23]. In addition, the inhibition of TGF-β signaling has been shown to reduce tumor growth and alleviate fibrosis in preclinical models of colorectal cancer [24], due to its trophic effect on tumor cells [25] and its profibrotic action [26]. Therefore, targeting the TGF-β signaling pathway with specific inhibitors could offer a promising strategy for developing new therapeutic options for human cancers [27].

Notably, in gastric cancer (GC), the overexpression of TGF-β and its receptor TGFBR1 serve as indicators of poor prognosis, especially for patients with high-grade tumors (grade 3) (Figure 1 and Figure 2). This underscores the potential of using TGF-β inhibitors, like SB431542, a selective TGFBR1 inhibitor, for targeted treatment of this group of patients. This approach is especially important given the current limitations in treatment options for grade 3 GC patients.

In the early phases of tumorigenesis, most cancer cells display sensitivity to TGF-β-mediated growth inhibition and apoptosis [28]. However, as the disease advances, TGF-β becomes a key driver of tumor progression through autocrine and paracrine signaling mechanisms that promote cellular invasion and metastasis. This dual function of TGF-β [29] suggests an initial tumor-suppressive role [30], which is later overridden as cancer cells acquire oncogenic mutations, enabling them to exploit TGF-β signaling for malignant progression [31]. Consequently, targeting TGF-β signaling pathways emerges as a promising strategy for therapeutic intervention [32].

In this work, we examined the biological effects of SB431542, a TGF-β signaling inhibitor that specifically targets TGFBR1 [33,34,35], on various aspects of tumorigenesis in three GC cell lines: AGS, SNU-1, and KATO III. Initially, a dose–response assay was performed to assess cell viability in the three cell lines exposed to different concentrations of SB431542 (1, 2, 5, 10, and 20 µM), to evaluate potential toxicity and sensitivity to the compound. Treatment with SB431542 at concentrations up to 20 µM did not significantly affect the viability of AGS cells. In contrast, SNU-1 cells exhibited a modest decrease in viability, while Kato III cells showed a more pronounced reduction in cell viability starting at 10 µM (Figure 3a–c). This differential response likely reflects the distinct genetic profiles of the cell lines. Specifically, AGS cells harbor mutations in *TGFBR1*, which impairs the normal receptor-ligand interaction. Additionally, AGS cells contain mutations in *SMAD4* and *RhoA* that are involved in TGF-β signaling. Consequently, these mutations may reduce the effectiveness of SB431542 in AGS cells. SNU-1 cells show resistance to the growth-inhibitory effects of TGF-β, likely due to a mutation in SMAD4, a critical component of the TGF-β signaling pathway. In contrast, KATO III cells lack mutations in key TGF-β signaling components, making them an appropriate control for assessing the effects of SB431542. (https://cancer.sanger.ac.uk/cell_lines, accessed on 30 July 2025).

We further performed a time-course experiment using different concentrations of the inhibitor to assess cell viability at various time-points of exposure. Treatment with 20 µM for 72 h resulted in a marked reduction in cell viability for SNU-1 and KATO III cells. At lower concentrations, particularly at 5 and 10 µM for 72 h, the decrease in viability was less pronounced in SNU-1 cells compared to KATO III cells, which exhibited a more marked reduction (Figure 4a–c). Therefore, being interested in studying the effect of SB431542 treatment on cells that demonstrated greater resistance, we performed the following experiments on AGS and SNU-1 cell lines. As a result, KATO III cells were excluded from further analyses.

5-fluorouracil is a widely used chemotherapeutic agent for gastric cancer treatment [36], often in combination with other drugs (e.g., FLOT regimen: 5-fluorouracil, leucovorin, oxaliplatin, docetaxel, in peri-operative therapy). Despite its clinical efficacy, 5FU therapy is frequently linked to substantial adverse effects, including nausea, fatigue, bone marrow suppression—resulting in decreased red blood cell, white blood cell, and platelet counts—alopecia, and gastrointestinal disturbances such as diarrhea [37,38]. In line with our previous studies [39], a high drug concentration was employed to confirm the effective resistance of the cell lines to the treatment.

5FU has well-documented cytotoxic effects on rapidly dividing normal cells, such as gastrointestinal and hematopoietic cells, consistent with its clinical side effects. However, its toxicity is significantly lower in quiescent or slow-proliferating normal cells. SB431542 has been reported to have no or minimal cytotoxicity in normal human cell lines, such as fibroblasts [40] and myoblasts [41] at micromolar concentrations. To the best of our knowledge, there are no published studies specifically addressing the cytotoxicity of the combination treatment of 5FU and SB431542 in normal human cell lines.

To investigate whether SB431542 could potentiate the toxic effects of chemotherapeutic agents thus allowing for a reduction in drug dosage and minimizing side effects in patients, we treated AGS and SNU-1 GC cells with 5FU. Notably, in both AGS and SNU-1 cell lines, the combination of 5FU and SB431542 induced significantly higher levels of cell death compared to 5FU alone. This enhanced cytotoxicity was observed not only at the highest dose of 5FU (200 μM), but also at intermediate concentrations (50 and 100 μM) and with different concentrations of SB431542 (10 and 20μM) (Figure 5 and Figure 6). These results indicate that inhibition of the TGF-β signaling pathway, which plays a role in promoting cellular survival and resistance to chemotherapy [29,42], can sensitize gastric cancer cells to 5FU treatment. Moreover, this combination strategy suggests that the effectiveness of chemotherapy could be improved with a lower dose of 5FU, potentially reducing the associated Undesirable effects and enhancing overall treatment efficacy.

To assess whether the effect on cell viability is mediated by alterations in caspase activation induced by the combination of 5FU and SB431542, we measured the activation of caspases III and VII, key markers of apoptosis [43], using a bioluminescence assay in a time-course experiment (8, 16, 24, 48, and 72 h). Notably, the activation of these caspases was significantly increased after the combinatorial treatment at both 24 and 48 h, supporting the hypothesis of a pro-apoptotic effect, especially potentiated by the inhibition of the TGF-β signaling pathway (Figure 7). To further confirm these results, we assessed the cleavage-mediated activation of caspases III and VII by Western blot analysis after 24 h of treatment. The data obtained were consistent with the luminescence findings, supporting the conclusion that the combination of 5FU and SB431542 promotes apoptosis through caspase activation via TGF-β pathway inhibition (Figure 8).

As an additional validation, cells were treated with Q-VD-Oph, a specific pan-caspase inhibitor. As expected, this treatment effectively reduced the pro-apoptotic effects induced by both 5FU and the 5FU + SB431542 combination, confirming that the observed cell death was caspase-dependent (Figure 9).

Finally, to assess the effect of SB431542 on cell proliferation, a colony formation assay was performed using AGS cells. AGS cells treated with 5FU alone demonstrated resistance, as indicated by their ability to form colonies following re-seeding at low density. However, in cells treated with the combination of 5FU and SB431542, no colonies were detected, indicating that the dual therapy not only suppressed cell proliferation but also diminished the cells’ ability to initiate tumorigenesis (Figure S5).

In conclusion, our data demonstrated that co-treatment with 5FU and SB431542 was especially effective in significantly reducing cell viability in GC cells as respect to 5FU alone. This combination therapy stands out because it does not only allow for a reduction in the required 5FU dosage, thereby minimizing the risk of toxicity associated with higher drug concentrations, but it also enhances the therapeutic effect of 5FU. Moreover, the co-treatment with SB431542 and 5FU resulted in a marked inhibition of colony formation, suggesting that the combination not only impairs the proliferation of GC cells but also disrupts their capacity to initiate tumorigenesis, a crucial factor in the metastatic potential of cancer. Furthermore, the findings from this study provide compelling evidence that SB431542, when combined with 5FU, could offer a novel approach to overcoming drug resistance in GC.

Resistance to chemotherapy remains a major challenge in treating GC, particularly in advanced stages. Targeting the TGF-β pathway could be an additional powerful strategy to sensitize tumors to existing chemotherapies, potentially enhancing treatment efficacy. This is especially relevant given the observed upregulation of TGF-β in grade 3 GC, a stage where therapeutic options are limited, and prognosis is poor. The increased TGF-β expression in grade 3 GC makes it an ideal candidate for targeted therapy with TGF-β inhibitors. Inhibiting the TGF-β signaling pathway may help reduce tumor progression, improve chemotherapy responsiveness, and potentially prevent metastasis.

This study supports the potential of including TGF-β inhibitors like SB431542 into treatment regimens for high-grade gastric cancer, offering a promising strategy to improve patient outcomes. With further research and clinical validation, such combination therapies could not only overcome resistance to standard treatments but also enhance survival rates and quality of life for patients with advanced gastric cancer.

## 4. Materials and Methods

### 4.1. Bioinformatics Analyses

GEPIA2 (http://gepia2.cancer-pku.cn/#index, accessed on 16 July 2025), Kaplan–Meier Plotter (https://kmplot.com/analysis/; accessed on 16 July 2025) and OncoDB (https://oncodb.org/; accessed on 16 July 2025) web servers [44,45,46] were used to analyze TGF-β and TGFBR1 expression in normal and tumor tissue samples, as well as its potential correlation with the prognosis of gastric cancer patients. In particular, for the GEPIA2 analysis, the TCGA-STAD (The Cancer Genome Atlas Stomach Adenocarcinoma) dataset was used to profile the tissue-specific expression of TGF-β and TGFBR1. To estimate overall and relapse-free survival in gastric cancer patients based on mRNA expression (RNA-seq) data, the Kaplan–Meier Plotter was employed. The selected cut-off value was “auto-selected best cut-off”. A *p*-value of <0.05 was considered statistically significant. The OncoDB web server was used to evaluate TGF-β and TGFBR1 expression in relation to different clinical parameters, such as tumor stage and histological grade.

### 4.2. Cell Lines

AGS, SNU-1 and KATO III gastric cancer cell lines, were from ATCC (American Type Culture Collection, Manassas, VA, USA). All cells were grown at 37 °C in 5% CO_2_. AGS were maintained as a sub confluent monolayer in Dulbecco’s modified Eagle’s medium (Euroclone, Milan, Italy) supplemented with 10% fetal bovine serum (Euroclone, Milan, Italy), 1% penicillin/streptomycin (P/S, Euroclone, Milan, Italy) and 1% Zell Shield (Minerva Biolabs GmbH, Berlin, Germany), SNU-1 were maintained in suspension in RPMI (Euroclone, Milan, Italy) supplemented with 10% fetal bovine serum, 1% P/S and 1% Zell Shield, and KATO III were maintained in part as floating cells and in part as layer cells in HyClone Iscove’s Modified Dulbecco’s Medium (IMDM) (GE HealthCare, Milan, Italy) supplemented with 20% fetal bovine serum, 1% P/S and 1% Zell Shield. Cells were routinely checked for mycoplasma contamination each time a new stock was thawed. SB4315425, a specific TGFBR1 inhibitor [47], (Selleckchem, Houston, TX, USA) and Q-VD-Oph, a pan inhibitor of caspase (Selleckchem, Houston, TX, USA) were dissolved in dimethyl sulfoxide, while 5-fluorouracil (5FU, Teva, Milan, Italy) was dissolved in sterile water.

### 4.3. Cell Viability Experiment

AGS, SNU-1 and KATO III cell lines were seeded in black 96-well plates in octuplicate to reach approximately 70% confluence after 24 h. Cells were then treated with increasing concentrations of SB431542 (0, 1, 2, 5, 10, 20 μM), 5FU (10−50−100−200 μM) or a combination of the two drugs. Results were compared with untreated cells (NT) as controls (Ctrl). Cell viability was evaluated after 72 h of treatment by Cell Titer Glo^®^ 2.0 assay (Promega, Milan, Italy) and the luminescence was read with a Tecan Infinite M200 pro (Tecan, Milan, Italy) luminometer.

### 4.4. Cell Growth/Proliferation Assay

AGS, SNU-1 and KATO III cell lines were seeded at a density of 5 × 10^3^ cells in black 96-well plates in octuplicate. Starting the following day (day 0), a proliferation assay was performed after treatment with increasing concentration of SB431542 (0, 1, 2, 5, 10, 20 μM). Proliferation was evaluated every 24 h for 72 h by an ATP-based assay (Cell Titer Glo^®^ 2.0, Promega, Milan, Italy) and the luminescence was read using Tecan Infinite M200 pro luminometer (Tecan, Milan, Italy).

### 4.5. Colony Assay

AGS cell line was plated in triplicate in 96-well plates to reach approximately 70% confluence after 24 h and then treated with 5FU 200 μM, SB431542 10 μM alone or a combination of both drugs. After 14 h of treatment, the cells were detached and seeded at low density (1500 cell/well) in triplicate in a 6-well plate for colony assay. Medium was replaced every three days and after 10–12 days colonies were fixed and stained in 0.5% crystal violet (Merk Life Science, Milan, Italy), 35% ethanol. Results were compared with untreated cells (NT) as ctrl. The quantification of the colonies was done using ImageQuant TL 8.0 software, an image analysis software for colony counting (Cytiva, Marlborough, MA, USA).

### 4.6. Caspase III–VII Assay

AGS and SNU-1 cell lines were seeded in quadruplicate in black 96-well plates to reach approximately 70% confluence after 24 h. Cells were then treated with 5FU 200 μM, SB431542 10 μM or 5FU 200μM + SB431542 10 μM. Analysis of Caspase activation was performed at different time-points (8, 16, 24, 48, and 72 h) by Caspase-Glo^®^ 3/7 Assay System (Promega, Milan, Italy). Results were compared with untreated cells (NT) as ctrl.

### 4.7. Western Blot Analyses

AGS and SNU-1 cell lines were seeded in triplicate in 6-well plates to reach approximately 70% confluence after 24 h and were then treated with 5FU 200 μM, SB431542 10 μM or 5FU 200 μM + SB431542 10 μM. Cell pellets were collected after 24 h of treatment.

Total protein extracts of these pellets were prepared using high-salt lysis RIPA buffer (Tris HCl 1.5 M, NaCl 5 M, sodium deoxicolate 10%, IGEPAL 1%) supplemented with 1% protease inhibitor cocktail (Sigma-Aldrich, St. Louis, MO, USA). Then, 20 μg of protein per sample was separated on 14% Tris Glycine gels (Invitrogen, Milan, Italy), and transferred onto a nitrocellulose membrane (Invitrogen, Milan, Italy) using iBlot system (Invitrogen) following manufacturer’s instructions. Membranes were blocked with 5% BSA (Merk Life Science, Milan, Italy) for 1 h and incubated at 4 °C overnight with the following primary antibodies: polyclonal rabbit anti-Full length Caspase 3 (1:1000 in BSA 5%) (Cell Signaling, Euroclone, Milan, Italy), polyclonal rabbit anti-Cleaved Caspase 3 (1:1000 in BSA 5%) (Cell Signalling, Euroclone, Milan, Italy) and polyclonal rabbit anti Total Caspase 7 (1:1000 in BSA 5%) (Cell Signalling, Euroclone, Milan, Italy). Immunoblotting experiments were repeated three times with consistent results. Each single blot was probed also with anti-actin antibody (Clone A1978, Sigma, Milan, Italy) as loading control. Images were acquired using a G_BOX XT4 Chemiluminescence and Fluorescence Imaging System (Syngene, Cambridge, UK) and bands were quantified with Image-J software 1.48v. Results were normalized expressed to β-actin and cleaved caspase levels were expressed respect their full-length form. Average ± SEM are plotted in the graphs.

### 4.8. Q-VD-Oph Treatments

AGS and SNU-1 cells were seeded in quadruplicate in black 96-well plates to reach approximately 70% confluence after 24 h. Cells were then treated with 5FU 200 μM, SB431542 10 μM, Q-VD-Oph 10 μM, 5FU 200 μM + SB431542 10 μM, 5FU 200 μM + Q-VD-Oph 10 μM, SB431542 10 μM + Q-VD-Oph 10 μM or 5FU 200 μM + SB431542 10 μM + Q-VD-Oph 10 μM. Cell viability was evaluated after 72 h by ATP-based assay (Cell Titer Glo^®^ 2.0, Promega, Milan, Italy) and the luminescence was read with Tecan Infinite M200 pro luminometer. Results were compared with untreated cells (NT) as ctrl.

### 4.9. Statistical Analysis

All data are expressed as the mean ± SEM Statistical significance was assessed using one-way ANOVA tests followed by Bonferroni and Dunnet post hoc tests for multiple comparisons, or Student’s *t*-test in other cases. All analyses were performed using the IBM SPSS statistics 28.0.0.0 software package (SPSS, Chicago, IL, USA). Differences were considered statistically significant at *p*-value ≤ 0.05.

## Data Availability

The original contributions presented in this study are included in the article/Supplementary Materials. Further inquiries can be directed to the corresponding author.

## Supplementary Materials

The following supporting information can be downloaded at https://www.mdpi.com/article/10.3390/ijms262311250/s1.

## Abbreviations

The following abbreviations are used in this manuscript:

5FU	5-fluorouracil
GC	Gastric Cancer
OS	Overall Survival
RFS	Relapse Free Survival
TGF-β	Transforming Growth Factor β
TGFBR1	Transforming Growth Factor β receptor 1
