# Supplementary material for: TGF-β Receptor Inhibitor SB431542 Enhanced the Sensitivity of Gastric Cancer to 5-Fluorouracil: New Combined Targeted Therapy
Source: Int J Mol Sci. 2025 Nov 21;26(23):11250. doi: 10.3390/ijms262311250 (PMC12692643; doi:10.3390/ijms262311250)
Supplement: Supplementary file 1 [file ijms-26-11250-s001.zip › Figure S1.pdf]

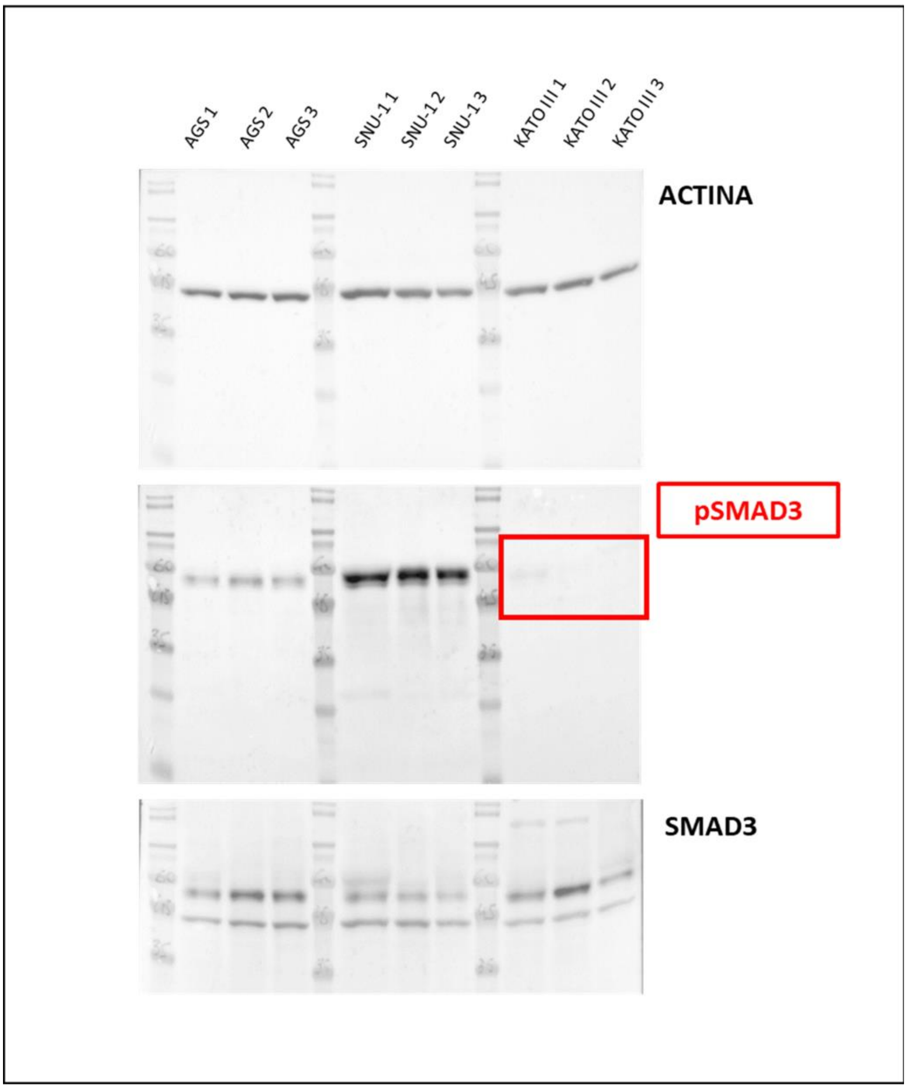

Figure S1: Expression of pSMAD3 and SMAD3 in AGS, SNU-1 and KATO III cell lines. Western blot analysis was performed on three independent protein extracts (1, 2, 3).
