# Supplementary material for: TGF-β Receptor Inhibitor SB431542 Enhanced the Sensitivity of Gastric Cancer to 5-Fluorouracil: New Combined Targeted Therapy
Source: Int J Mol Sci. 2025 Nov 21;26(23):11250. doi: 10.3390/ijms262311250 (PMC12692643; doi:10.3390/ijms262311250)
Supplement: Supplementary file 1 [file ijms-26-11250-s001.zip › Figure S2.pdf]

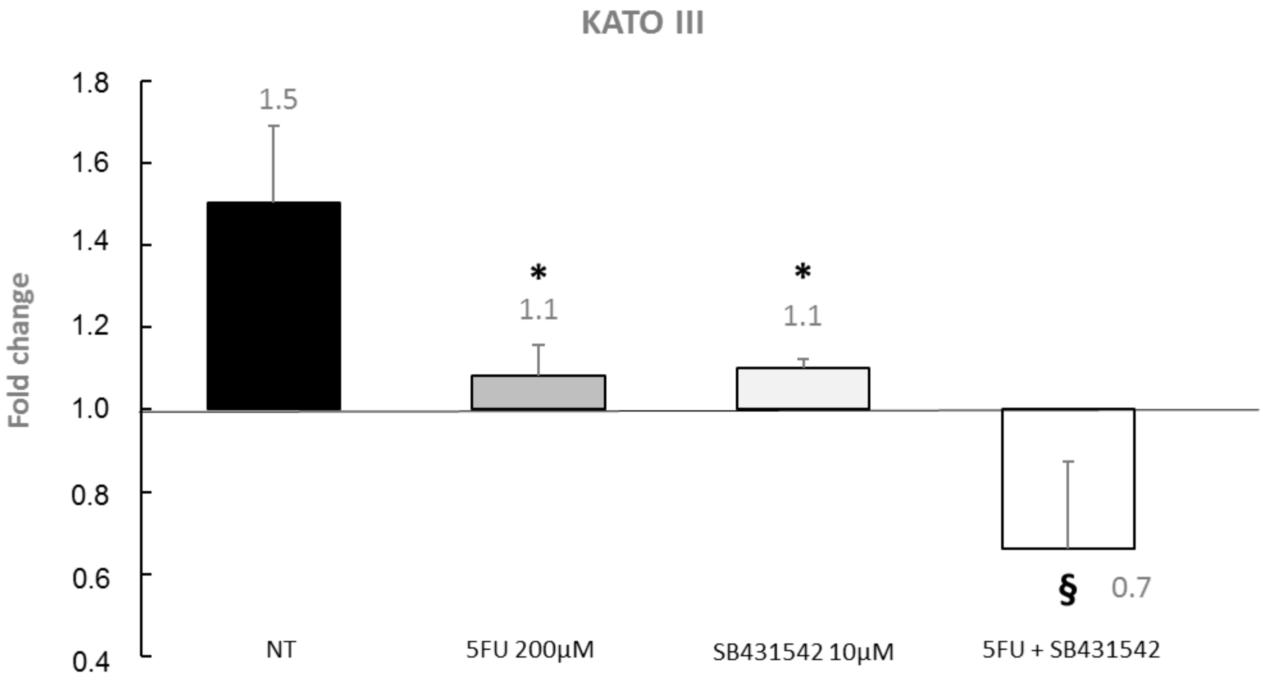

Figure S2: Cell viability experiments with 5FU (200 µM) or SB431542 (10 µM) alone or in combination on KATO III cells. Cell viability was assessed by the Cell Titer Glo Assay after 72h of treatments. NT: untreated cells. The graphs represent the mean of three separate experiments. Mean ± SEM are plotted in the graphs. p-value < 0.05 was considered statistically significant (* $p < 0.05$ vs. NT; § $p < 0.05$ vs. both NT and 5FU).
