# Supplementary material for: TGF-β Receptor Inhibitor SB431542 Enhanced the Sensitivity of Gastric Cancer to 5-Fluorouracil: New Combined Targeted Therapy
Source: Int J Mol Sci. 2025 Nov 21;26(23):11250. doi: 10.3390/ijms262311250 (PMC12692643; doi:10.3390/ijms262311250)
Supplement: Supplementary file 1 [file ijms-26-11250-s001.zip › Figure S4.pdf]

Exp. 1

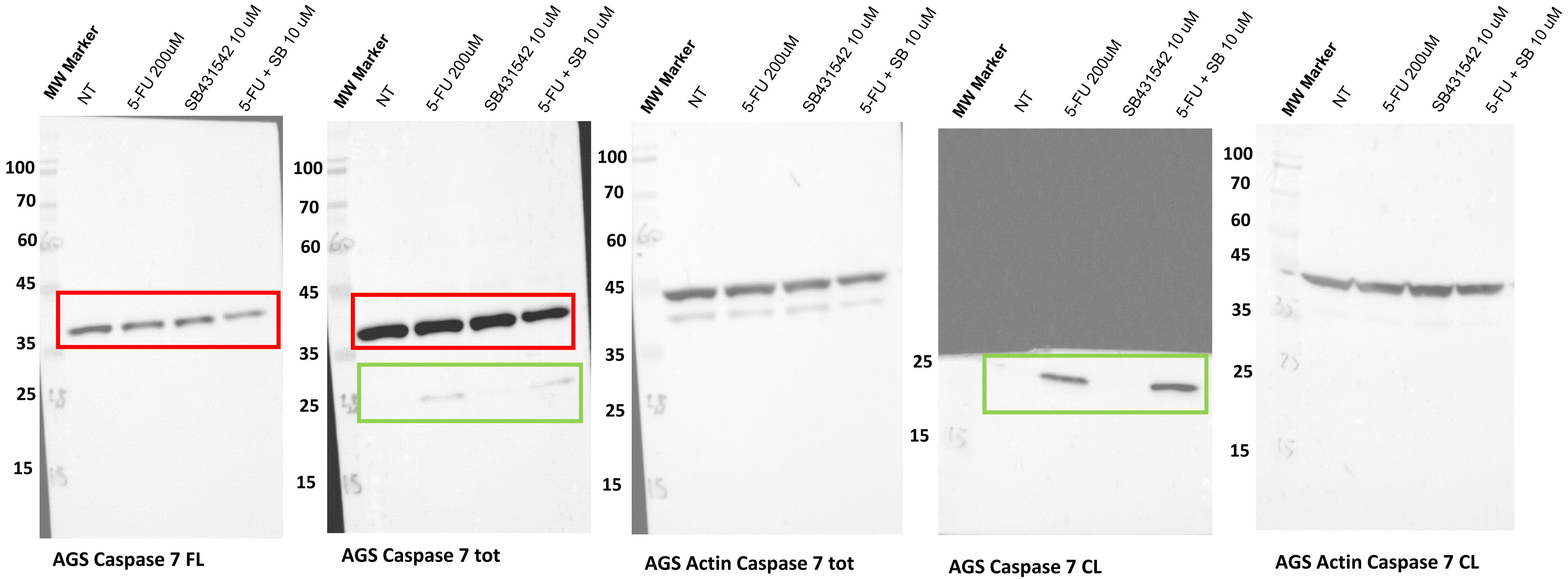

Ref. Figure 8a

Exp. 1

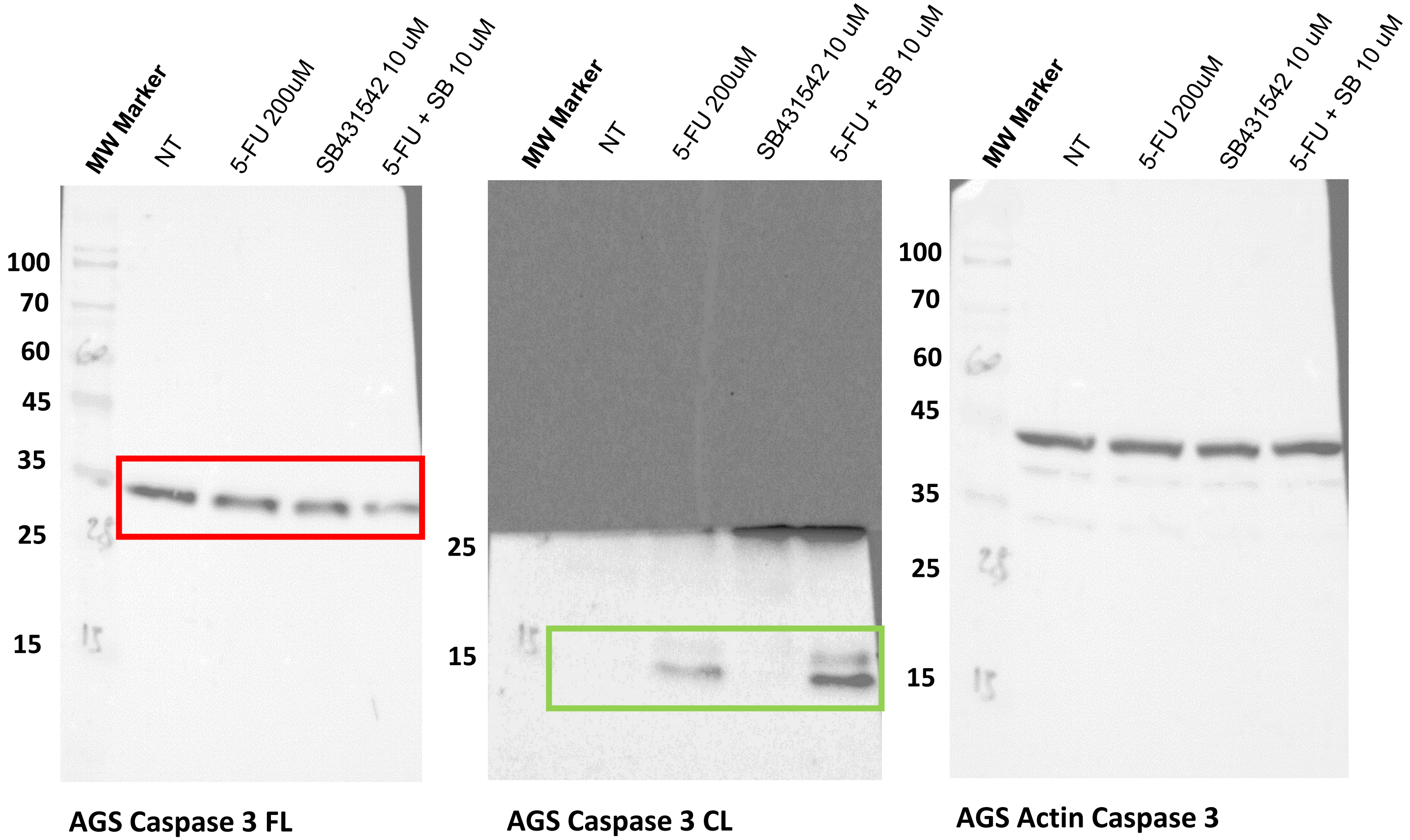

Ref. Figure 8a

Exp. 1

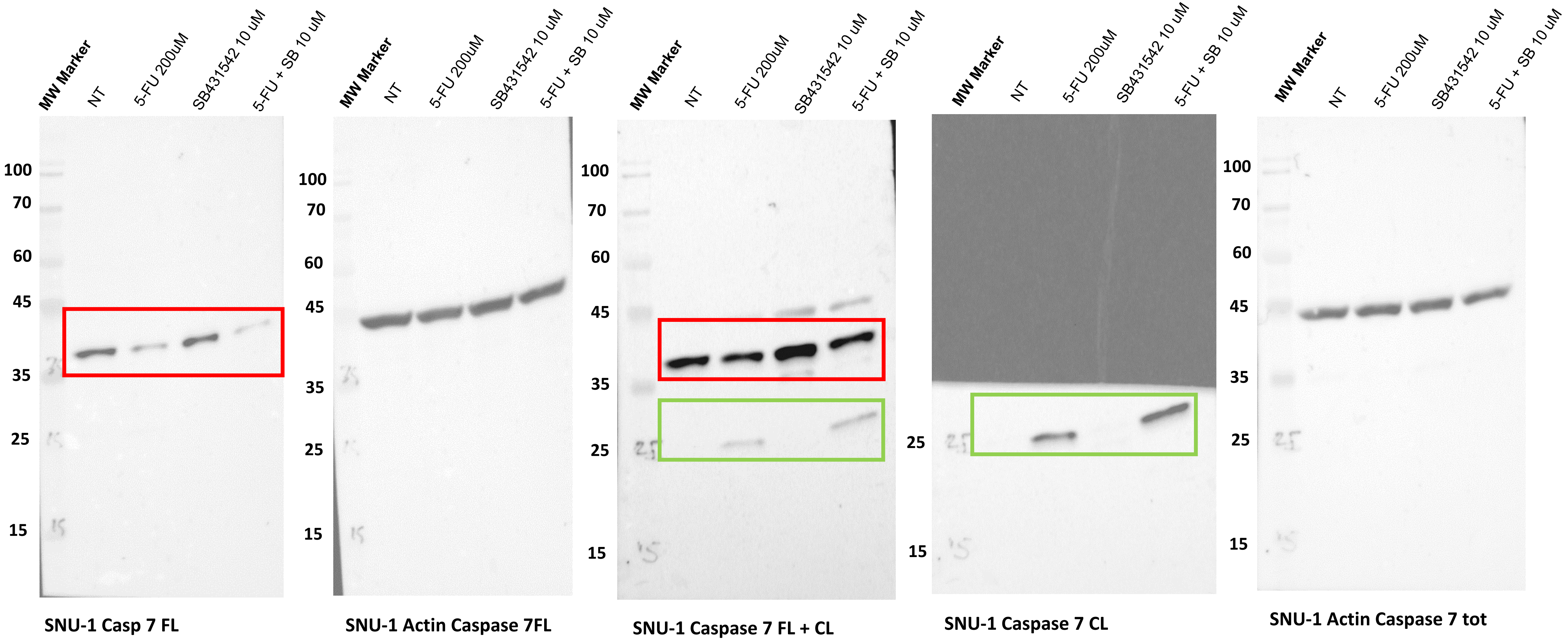

Ref. Figure 8b

Exp. 1

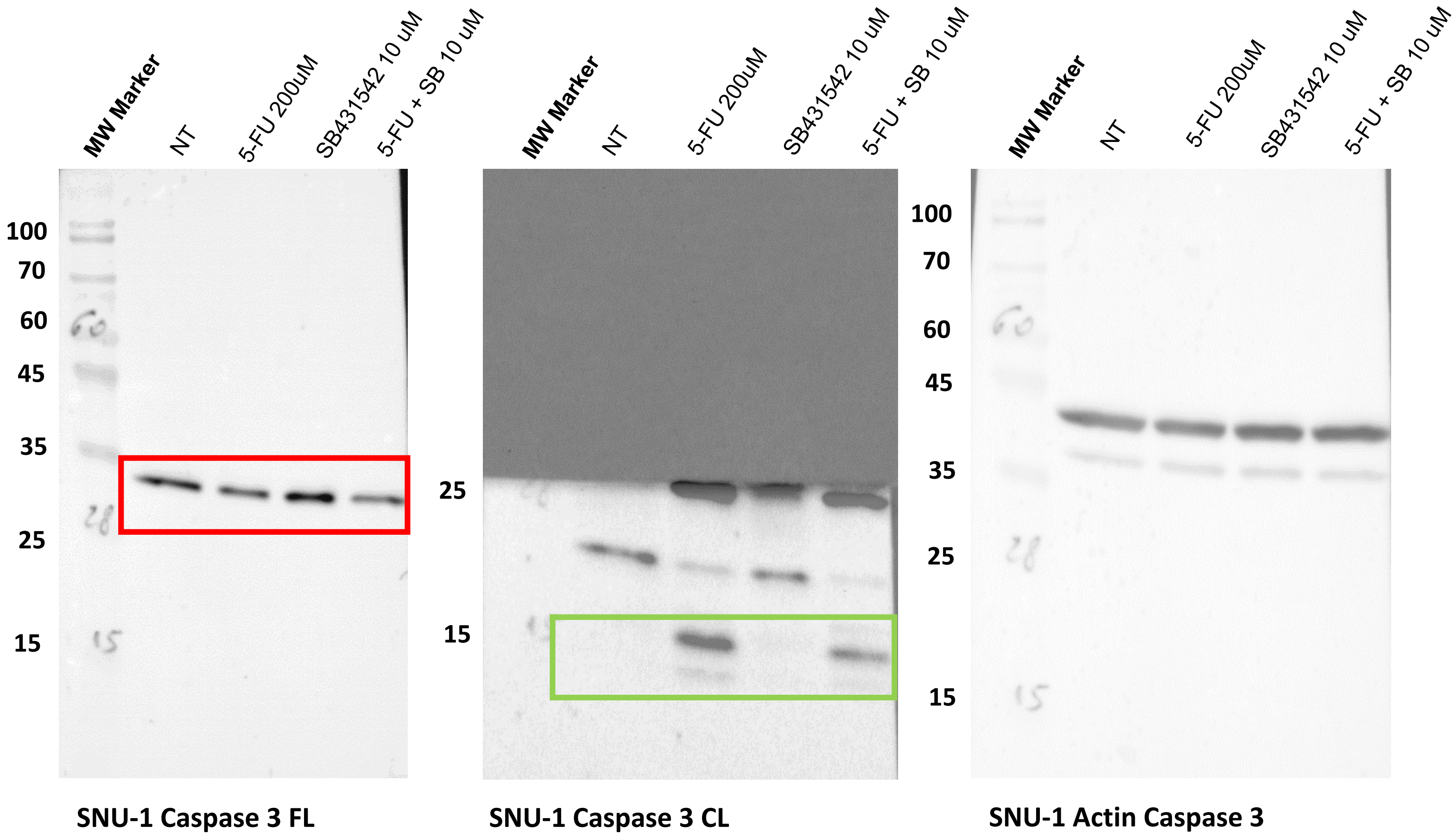

Ref. Figure 8b

Exp. 2

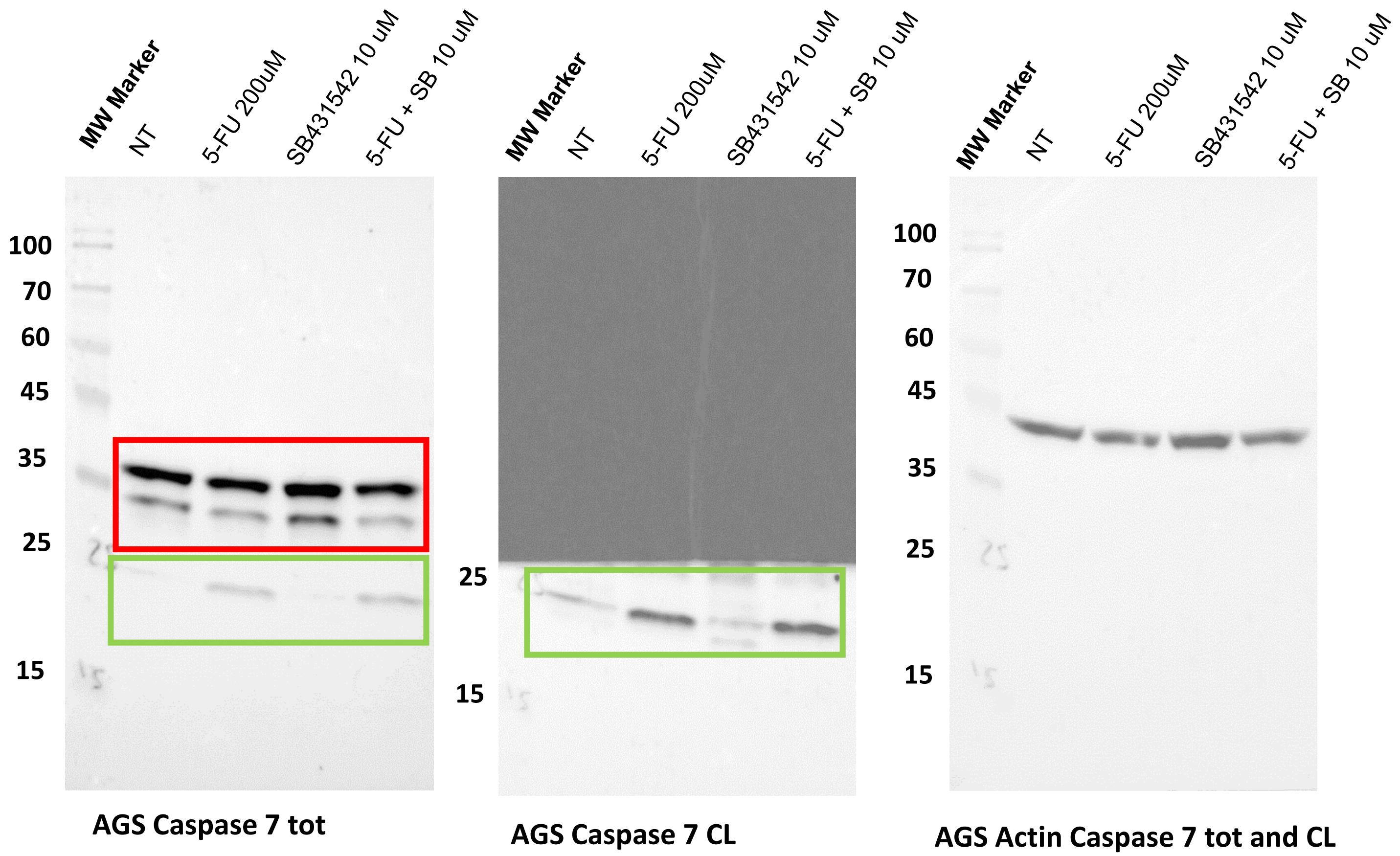

Ref. Figure 8a

Exp. 2

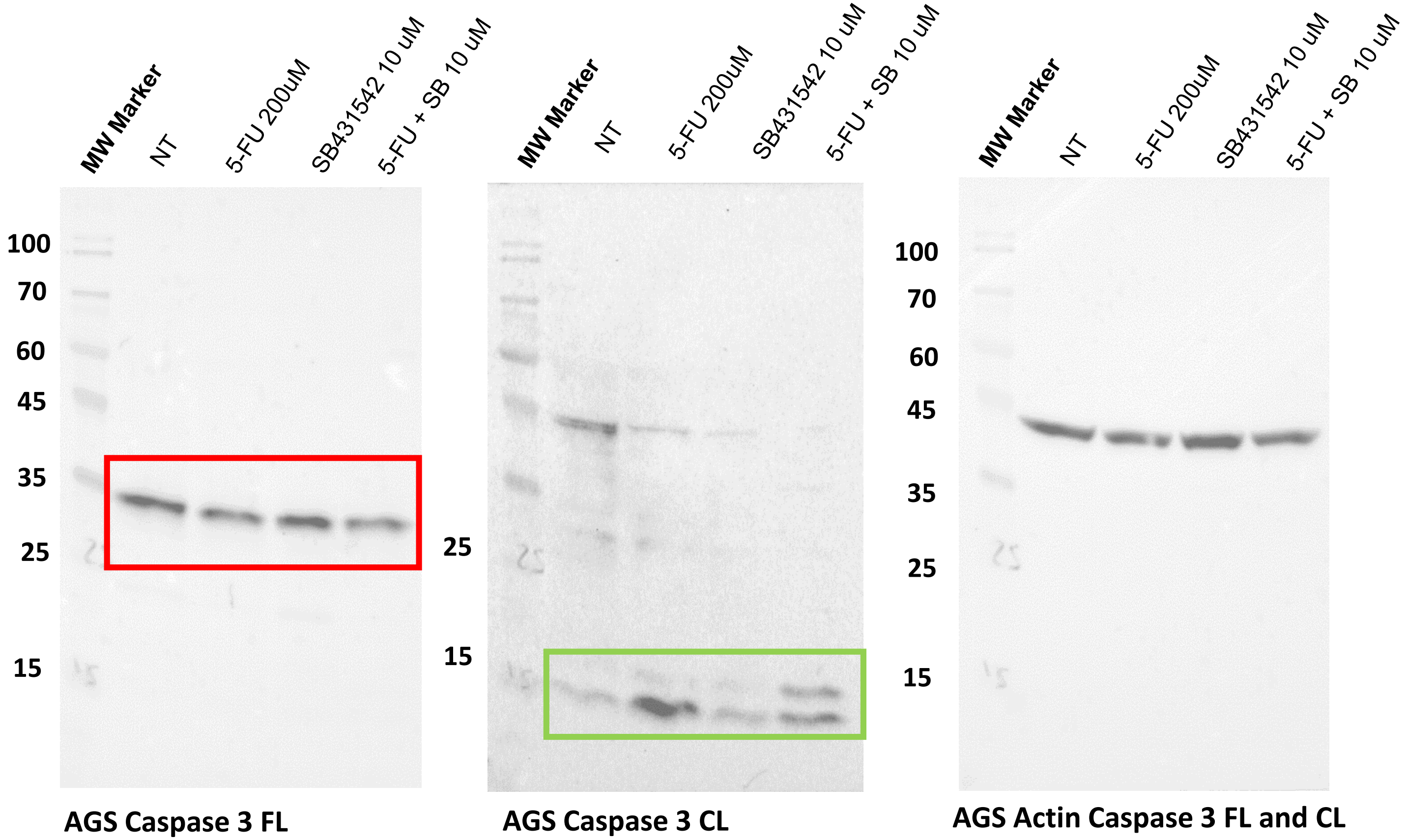

Ref. Figure 8a

Exp. 2

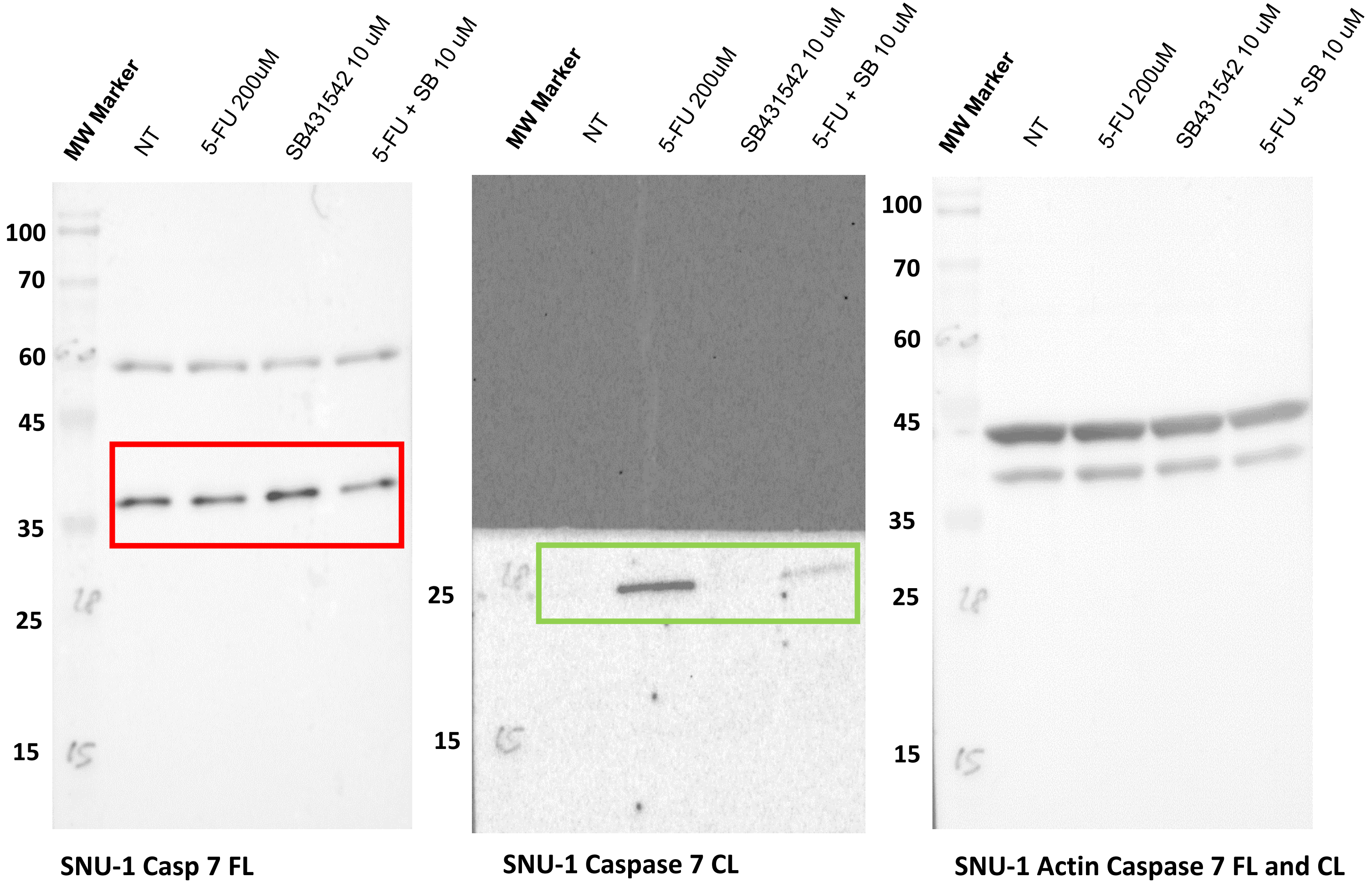

Ref. Figure 8b

Exp. 2

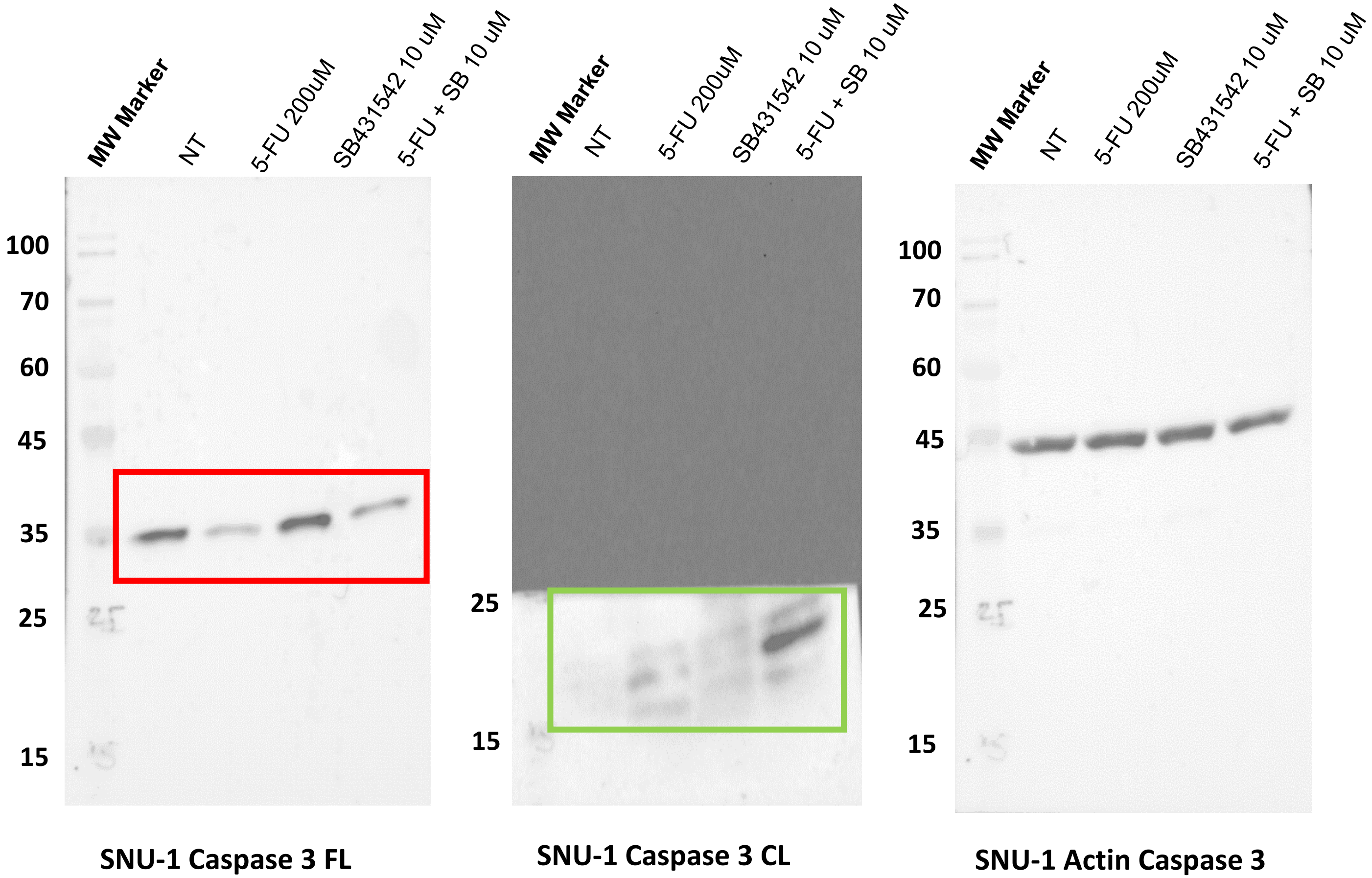

Ref. Figure 8b

Exp. 3

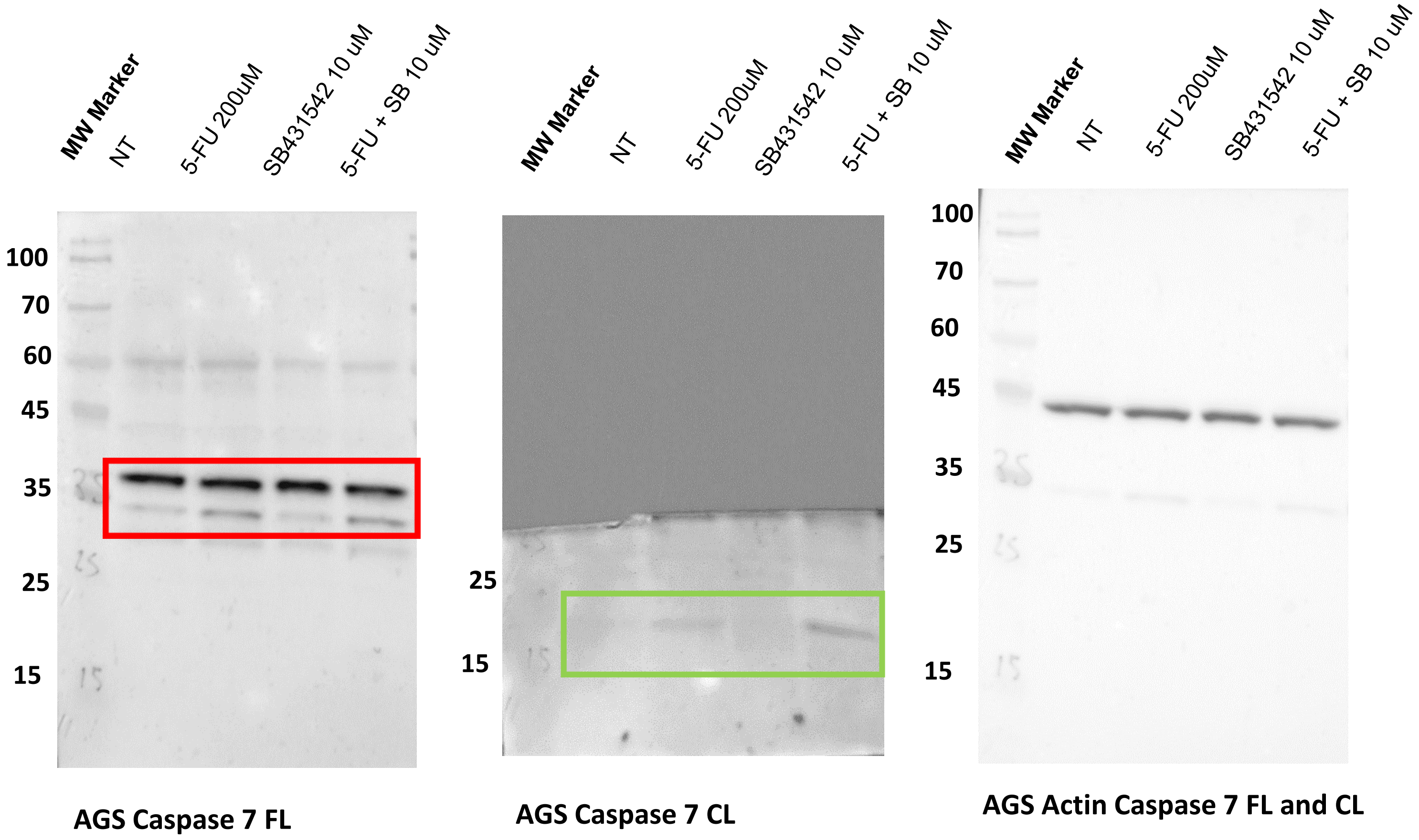

Ref. Figure 8a

Exp. 3

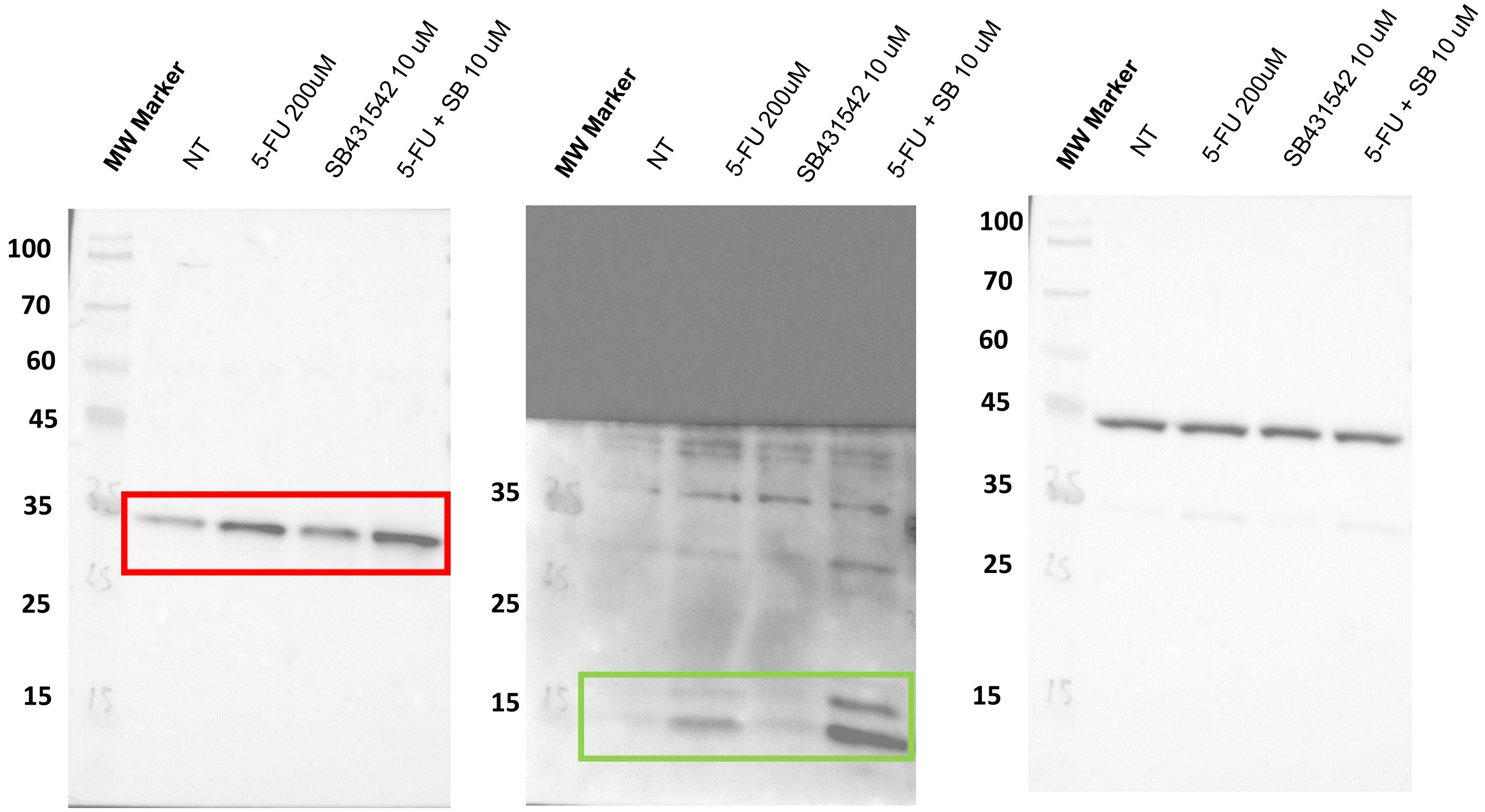

AGS Caspase 3 FL

AGS Caspase 3 CL

AGS Actin Caspase 3 FL and CL

Ref. Figure 8a

Exp. 3

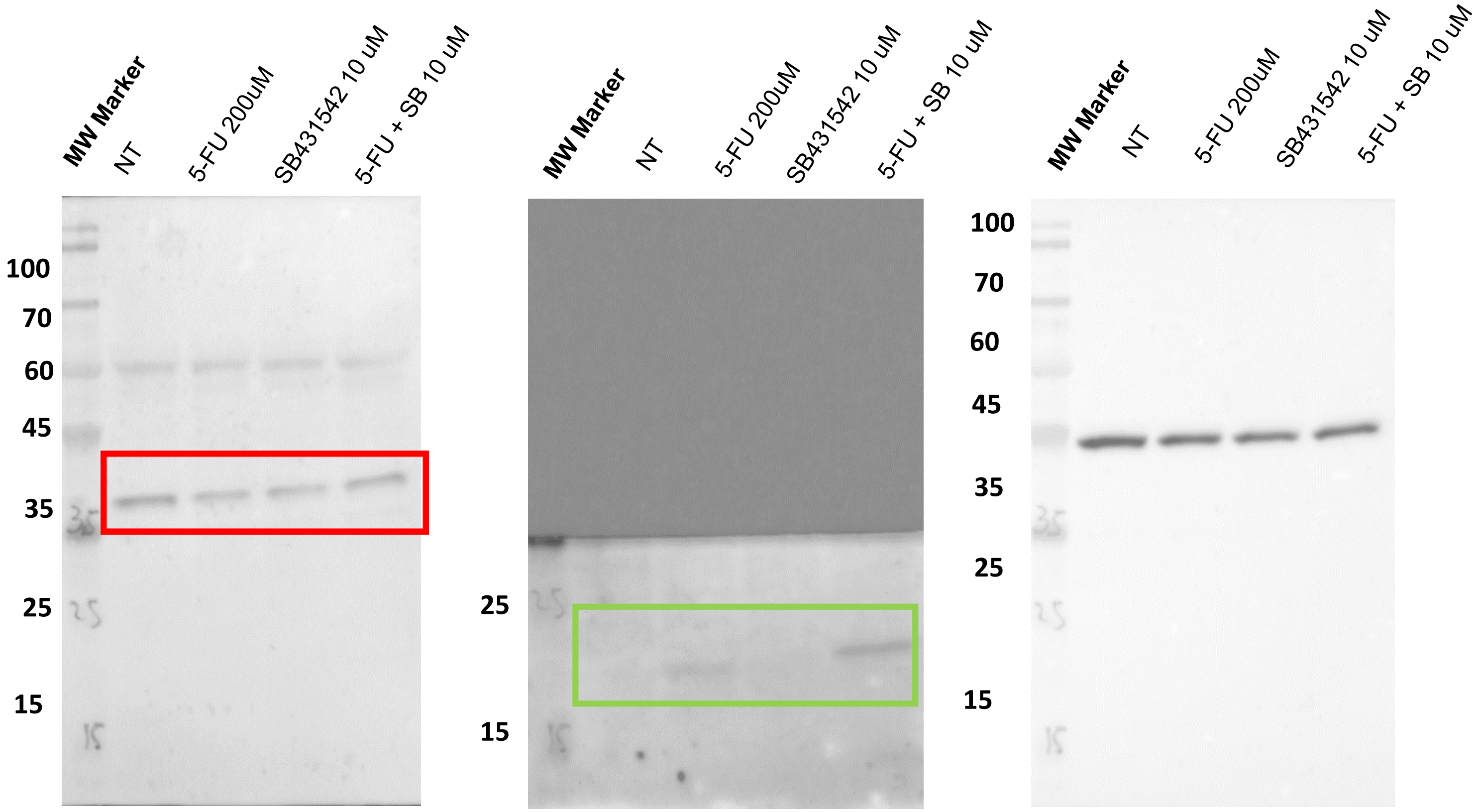

SNU-1 Caspase 7 FL

SNU-1 Caspase 7 CL

SNU-1 Actin Caspase 7 FL and CL

Ref. Figure 8b

Exp. 3

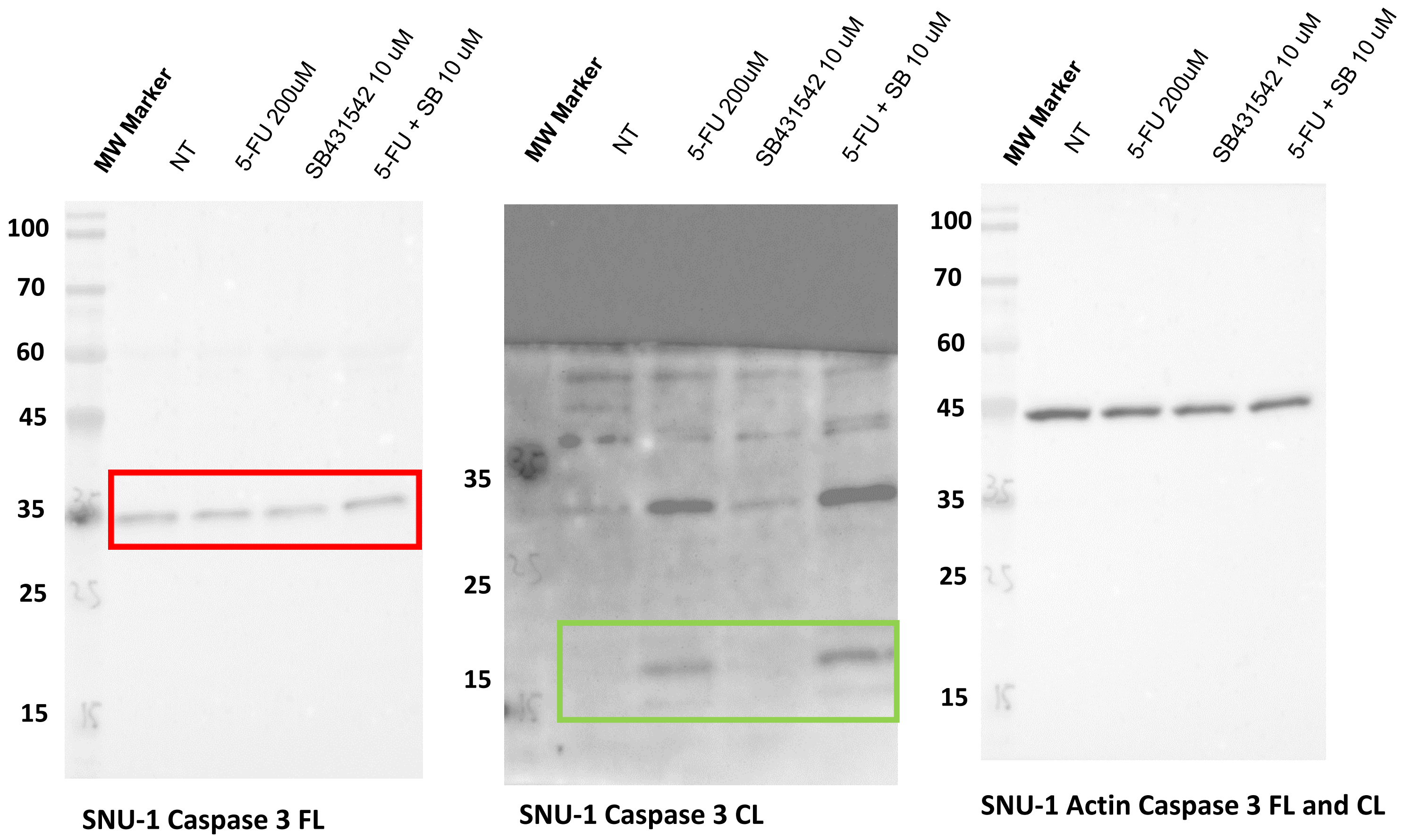

Figure S4: Full Western blot images from the three experiments reported in the paper for caspase-3 and caspase-7, full-length (FL) and cleaved (CL)

Ref. Figure 8b
