# Supplementary material for: TGF-β Receptor Inhibitor SB431542 Enhanced the Sensitivity of Gastric Cancer to 5-Fluorouracil: New Combined Targeted Therapy
Source: Int J Mol Sci. 2025 Nov 21;26(23):11250. doi: 10.3390/ijms262311250 (PMC12692643; doi:10.3390/ijms262311250)
Supplement: Supplementary file 1 [file ijms-26-11250-s001.zip › Figure S5.pdf]

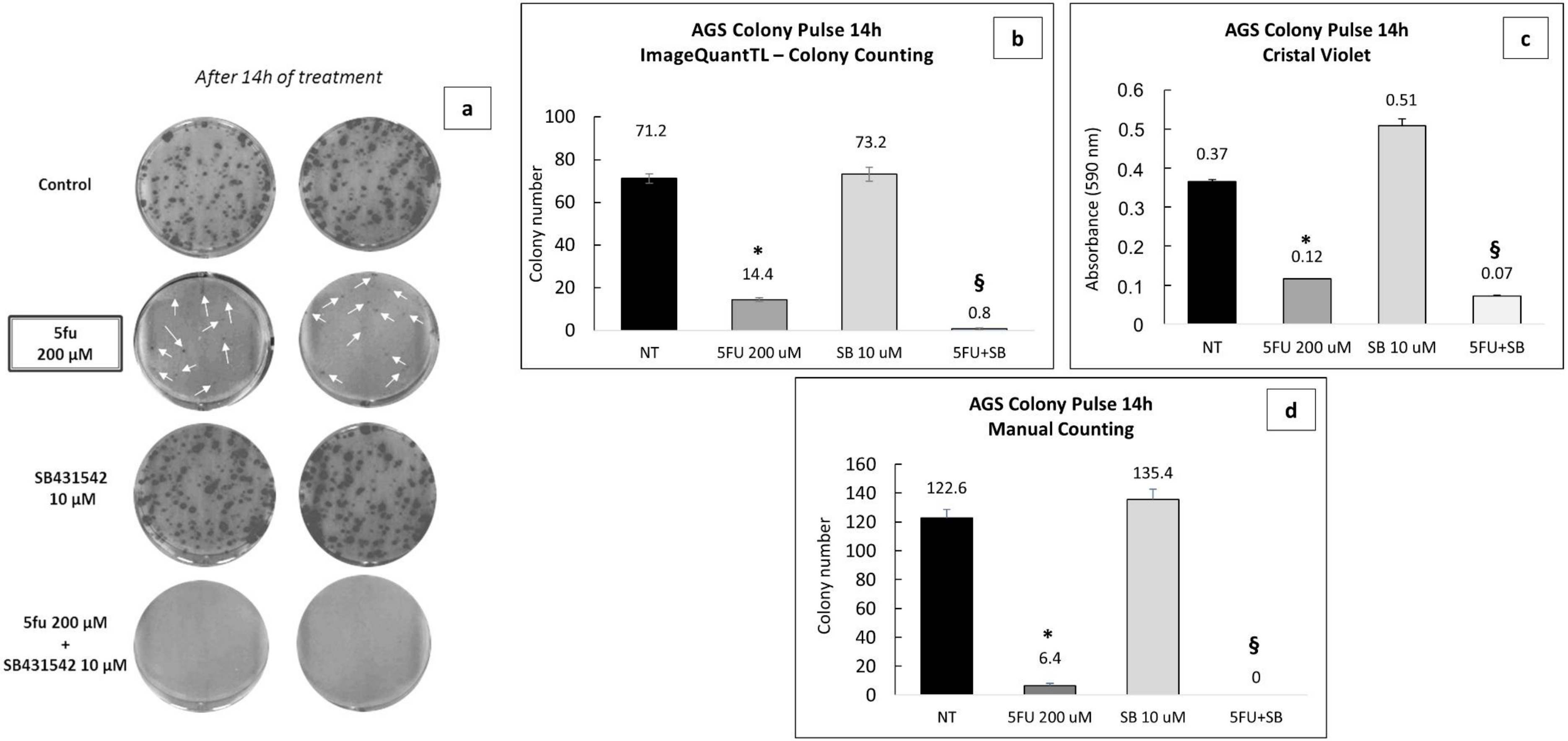

Figure S5: Clonogenic assay in the AGS cells previously treated with 5FU 200 µM, SB431542 10 µM or 5FU 200 µM + SB431542 10 µM. Colonies were allowed to grow for 12 days and then were stained with crystal violet. a) Representative images of colonies. White arrows highlight the colony growth. b) Quantification of colony formation using ImageQuant TL software (Cytiva). c) Colony assay quantification by resuspending crystal violet with 10% acetic acid and absorbance reading at 595 nm. d) Manual colony count. Mean ± sem are plotted in the graphs; data are the average of three independent experiments. p-value < 0.05 was considered statistically significant (* $p<0.05$ vs NT, § $p<0.05$ vs both NT and 5FU).
